# Mediating effect of vascular calcification in galectin-3-related mortality in hemodialysis patients

**DOI:** 10.1038/s41598-024-51383-2

**Published:** 2024-01-10

**Authors:** Ji-Hwan Kim, Hye-Mi Noh, Hong Ji Song, Sion Lee, Sung Gyun Kim, Jwa-Kyung Kim

**Affiliations:** 1 Department of Internal Medicine and Kidney Research Institute, Hallym University Sacred Heart Hospital, Pyungan-dong, Dongan-gu, Anyang, 431-070 Korea; 2 Department of Family Medicine, Hallym University Sacred Heart Hospital, Anyang, Korea; 3 Department of Statistics and Institute of Statistics, Hallym University, Chuncheon, Korea

**Keywords:** Cardiovascular biology, Nephrology, Predictive markers

## Abstract

Galectin-3 levels have been studied as a potential biomarker for predicting cardiovascular (CV) risk and mortality in hemodialysis (HD) patients. Recently, a close relationship between galectin-3 and vascular calcification (VC) has been reported. Here, we investigated the role of VC as a mediating factor in the association between galectin-3 and mortality. Serum galectin-3 and baseline aortic arch calcification (AoAC) score were measured in 477 incident HD patients. Mortality data were obtained at a median follow-up of 40 months. Causal mediation analysis was performed to examine the effect of vascular risk factors on galectin-3-related mortality. The prevalence of AoAC in HD patients was 57% (n = 272), and elevated galectin-3 levels were associated with a significantly increased risk of AoAC. When the galectin-3 level was divided by the median level of 37 ng/mL, a higher galectin group increased the risk of all-cause mortality by 1.71-fold (95% CI 1.02–2.92, p = 0.048), even after adjustment for multiple CV risk factors. Mediation analysis showed that both the direct effect of the galectin-3 on mortality (β = 0.0368, bootstrapped 95% CI [0.0113–0.0622]) and the indirect effects were significant. AoAC score and high-sensitivity CRP levels significantly mediated the association between galectin-3 and mortality (total indirect effects: β = 0.0188, bootstrapped 95% CI [0.0066–0.0352]). This study suggests that the association between high galectin-3 and mortality may be partially mediated by higher VC and inflammatory state in HD patients.

## Introduction

Cardiovascular disease (CVD) is the leading cause of death in dialysis patients^1^, and previous studies have reported that vascular calcification (VC) increases the risk of cardiovascular (CV) or all-cause mortality in the elderly general population as well as in dialysis patients^2,3^. VC is a pathological condition involving phenotypic differentiation of vascular smooth muscle cells (VSMCs) into myofibroblasts, resulting in the deposition of bone-specific hydroxyapatite crystals on the vessel wall. It is one of the most important features representing the severity of atherosclerosis and is therefore used for CV risk stratification and as a prognostic marker in chronic kidney disease (CKD) and hemodialysis (HD) patients^4^. Abdominal lateral or chest radiographs are commonly performed as a routine screening test for VC in dialysis patients, and physicians can easily measure the abdominal aortic calcification (AAC) or aortic arch calcification (AoAC) score. The prevalence of AoAC in dialysis patients is commonly reported to be between 40.7 and 58%^3^.

Galectin-3 is a β-galactoside-binding protein that is widely expressed in various tissues^5^. Functionally, galectin-3 can be located intracellularly or secreted into the extracellular space as a soluble protein that plays an important role in cell processes such as cell proliferation, differentiation, apoptosis, fibrosis, and inflammation. Recent studies have implicated galectin-3 in many chronic diseases, including coronary artery disease (CAD), heart failure (HF), CKD, and atrial fibrillation^6–8^. Particularly in CKD, the levels of galectin-3 levels increase with the deterioration of renal function and are significantly elevated in patients with HD^9^. A recent meta-analysis showed that galectin-3 is associated with an increased risk of all-cause mortality and CV events in this population^10,11^. In addition, galectin-3 has been implicated in the pathogenesis of atherosclerosis^12^. The beneficial effects of reducing galectin-3 on lesion size in apolipoprotein E-deficient mice have been attributed mainly to the proinflammatory properties of galectin-3^13,14^.

More recently, it has also been suggested that galectin-3 may be involved in the regulation of vascular inflammation and osteogenesis, thus playing an important role in the development and progression of VC^15^. It can directly cause intimal calcification by promoting osteogenic differentiation of VSMC and extracellular remodeling through several signaling pathways, such as Wnt/β-catenin signaling pathway, NF-κB signaling pathway, and ERK1/2 signaling pathway^9,16^. A prospective cohort study of abdominal aortic calcification in HD patients by Wang et al. showed that serum galectin-3 was an independent risk factor for severe AAC and AAC progression^17^.

Despite the recent evidence of the close association between galectin-3, VC, and mortality risk, few studies have investigated the mediated effects of galectin-3 and VC on mortality in HD patients. In this regard, we hypothesized that increased VC will be identified as a mediating factor in the association between high galectin-3 and mortality. Therefore, this study aims to determine whether (1) high galectin-3 levels may affect AoAC in incident HD patients and (2) how these effects may contribute to the risk of mortality in this population.

## Methods

### Study population and sampling

We prospectively enrolled new stable HD patients from January 2012. Patients who started HD because of acute kidney injury and those with unstable vital signs (starting continuous renal replacement therapy) were not included. As a result, a total of 523 incident HD patients was enrolled. The last enrollment was done in February 2022. During follow-up, we tried to contact the patients regularly through outpatient clinic visits, even if they were transferred to another hospital or long-term care facility. However, 46 patients were lost to follow-up during the first 3 months of HD. Therefore, 477 patients were analyzed. During the follow-up, 40 patients received kidney transplant and these cases were censored. This study was approved by the Institutional Review Board/Ethics Committee of Hallym University Sacred Heart Hospital, Anyang, Korea, and conducted in accordance with the Declaration of Helsinki (approval number: 2015-I113). A written informed consent was obtained from each patient. Baseline demographic data, including age, sex, comorbidities, and clinical data regarding the underlying cause of renal disease were obtained. Baseline BMI was calculated as body weight (kg)/(height (cm)/100)^2^. All patients received regular HD 2–3 times per week on a 3.5–4 h schedule.

Venous sampling was performed immediately prior to each patient’s first HD session. Biochemical analyses of white blood cells (WBCs), neutrophils, lymphocytes, platelets, and levels of hemoglobin, serum albumin, calcium (Ca), phosphorus (P), total cholesterol, low density lipoprotein cholesterol (LDL-C), high-density lipoprotein cholesterol (HDL-C), triglyceride, blood urea nitrogen (BUN), creatinine, and high sensitivity C-reactive protein (hsCRP) were measured. In addition, CaxP product and neutrophil/lymphocyte ratio (NLR) were calculated. Serum B-natriuretic peptide (BNP) was measured at the time of first HD.

Serum galectin-3 was measured from 477 HD patients and 40 age- and sex- matched healthy volunteers using ELISA kits from R&D Systems (Minneapolis, MN, USA), according to the manufacturer’s instruction.

### Aortic arch calcification grading

The extent of AoAC was assessed in a routine posterior-anterior chest X-ray. The level of AoAC was categorized into four levels according to the categorization proposed in a previous report^18,19^. Briefly, we scored the area of calcification as four grades: grade 0, no visible calcification; grade 1, small spots of calcification or a single thin area of calcification of the aortic knob; grade 2, one or more areas of thick calcification less than 50%; grade 3, thick circular calcification of the aortic knob more than 50%. Two independent observers (Kim JH, Noh HM) blindly reviewed the chest radiographs of all subjects, and the Cohen’s kappa coefficient between the two staffs on the rate of agreement was 0.62 (p < 0.001).

### Endpoint

The study endpoint was all-cause and CV mortality. Because this study was based on a well-managed HD cohort, the cause of death was well-determined. CV mortality was defined as death due to myocardial ischemia and infarction (MI), heart failure, sudden cardiac arrest due to unknown cause, or cerebrovascular accident. Sudden cardiac death was considered CV mortality. For patients without mortality, the last patient contact was recorded until February 2023.

### Statistical analysis

Variables with normal distributions were reported as mean ± standard deviation (SD), and non-normal variables were reported as median with interquartile ranges. Receiver operating characteristic (ROC) curves were plotted for galectin-3 and BNP, to identify a cutoff for predicting mortality. Patients were divided according to the cutoff level, and the differences between the groups were determined by independent *t*-test and analysis of variance for continuous variables or the χ^2^ test for categorical data. The effect of high galectin-3 levels on higher AoAC score and all-cause mortality was assessed using logistic regression analysis and Cox proportional hazards model, respectively. Next, we performed causal mediation analysis to investigate the effect of galectin-3 (independent variable) on mortality (dependent variable) through AoAC score, HDL-C, BNP and hsCRP levels (third variable, called a mediator). The bootstrap procedure was used to determine the significance of the mediated effects. We determined 95% CIs from the 5000 bootstrap resamples, and any interval that did not include 0 was considered to be statistically significant. All analyses were performed using IBM SPSS Statistics for Windows version 28.0 (IBM Corp., Armonk, NY, USA) and R version 4.1.0. A p-value < 0.05 was considered significant.

### Ethics declarations

This study was approved by our institutional ethics committee and conducted in accordance with the Declaration of Helsinki (approval number: 2015-I113) and a written informed consent was obtained from each patient.

## Results

### Baseline characteristics

**Figure 1:**
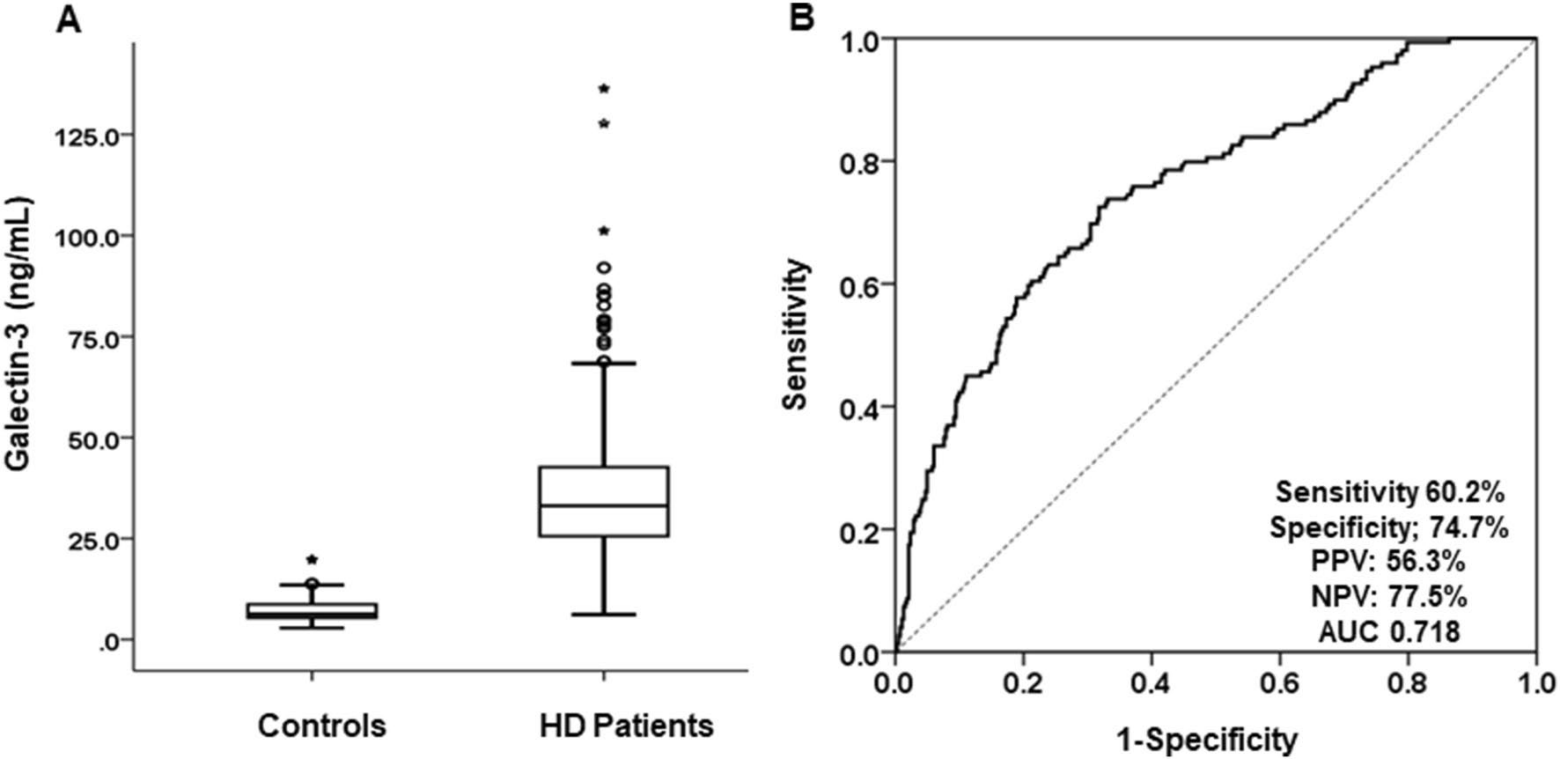
(**A**) Comparison of serum galectin-3 levels between HD patients and healthy controls. The mean serum galectin-3 levels of HD patients were significantly higher than those of healthy controls (p < 0.001). (**B**) Receiver operating characteristic curve (ROC) analysis of the prediction of mortality by serum galectin-3 levels. At a cut-off value of 37.0 ng/mL of serum galectin-3, the AUC for predicting mortality was 0.718 with sensitivity and specificity of 60.2% and 74.7%, respectively. *PPV* positive predictive value, *NPV* negative predictive value, *AUC* area under the ROC curve.

The baseline characteristics of the 477 patients who newly started HD are shown in Table 1. The mean age was 68.5 ± 12.6 years; 58.1% (n = 277) were men and more than half of the patients were aged ≥ 70 years (n = 244, 51.2%). Diabetic kidney disease was the most common cause of ESRD (n = 285, 59.7%) and 21.0% of patients (n = 100) had a previous history of CAD. As expected, serum galectin-3 levels were significantly higher in HD patients compared to healthy controls (35.8 ± 15.9 vs. 7.2 ± 2.9 ng/mL, p < 0.001) (Fig. 1A). In HD patients, ROC curve analysis revealed a strong association between serum galectin-3 levels and mortality. At a cut-off value of 37.0 ng/mL, the AUC for predicting mortality was 0.718 with sensitivity and specificity of 60.2% and 74.7%, respectively (Fig. 1B).

**Table 1:** Baseline characteristics of study subjects.

Variables	Total (n = 477)	Galectin-3		
		< 37.0 ng/mL (n = 284, 59.5%)	≥ 37.0 ng/mL (n = 193, 40.5%)	p
Age (years)	68.5 ± 12.6	67.0 ± 12.1	70.4 ± 13.2	0.004
≥ 70 years, n (%)	244 (51.2)	124 (43.7)	120 (62.2)	< 0.001
Gender, male, n (%)	277 (58.1)	171 (60.2)	106 (54.9)	0.146
Diabetes, n (%)	300 (63.0)	177 (62.5)	123 (63.7)	0.483
Coronary artery disease, n (%)	100 (21.0)	50 (17.7)	50 (25.9)	0.021
SBP, mmHg	140.3 ± 21.8	140.0 ± 22.6	140.7 ± 20.8	0.772
DBP, mmHg	78.2 ± 11.8	76.0 ± 12.2	78.9 ± 9.8	0.027
BMI (kg/m^2^)	24.7 ± 4.3	24.7 ± 4.2	24.8 ± 4.5	0.974
WBC (/μL)	6876 ± 1812	6676 ± 1720	7160 ± 1904	0.009
Hemoglobin (g/dL)	9.3 ± 1.7	9.4 ± 1.6	9.3 ± 1.7	0.750
Neutrophil, (/μL)	4795 ± 1689	4531 ± 1585	5170 ± 1764	< 0.001
Neutrophil/Lymphocyte	4.5 ± 3.2	4.1 ± 2.9	5.0 ± 3.6	0.009
Platelet (× 10^3^/μL)	193 ± 75	190 ± 75	200 ± 75	0.244
BUN, mg/dL	77.6 ± 30.4	78.0 ± 29.6	77.0 ± 31.9	0.800
Creatinine, mg/dL	7.2 ± 3.0	7.1 ± 2.9	7.3 ± 3.2	0.726
Serum calcium (mg/dL)	8.1 ± 0.9	8.1 ± 0.9	8.2 ± 0.9	0.462
Serum Phosphate (mg/dL)	5.0 ± 1.6	4.9 ± 1.5	5.1 ± 1.8	0.279
Ca * P	40.4 ± 12.9	39.7 ± 11.1	41.3 ± 14.6	0.256
25(OH) vitamin D (ng/mL)	8.8 ± 6.5	8.9 ± 6.2	8.6 ± 6.9	0.692
Total cholesterol (mg/dL)	153.5 ± 46.9	155.0 ± 45.2	151.4 ± 49.4	0.441
Triglyceride (mg/dL)	138.0 ± 91.4	134.2 ± 97.9	143.6 ± 80.9	0.335
HDL cholesterol, mg/dL	43.8 ± 14.6	45.6 ± 15.0	41.1 ± 13.7	0.004
LDL cholesterol, mg/dL	93.9 ± 36.5	92.3 ± 30.6	96.2 ± 43.7	0.312
Albumin (g/dL)	3.5 ± 1.7	3.5 ± 0.5	3.6 ± 2.5	0.611
BNP (mmol/L)*	562 (174- 1489)	421 (150–1195)	848 (239–1724)	< 0.001
hsCRP* (mg/L)	1.10 (0.50–3.03)	0.88 (0.39–2.26)	1.60 (0.82–4.23)	< 0.001
Anti-hypertensive agents, n (%)	408 (85.7)	240 (84.8)	168 (87.0)	0.291
RAS blocker, n (%)	346 (72.5)	206 (71.4)	140 (72.5)	0.326
Ca-based P binder, n (%)	171 (35.8)	98 (34.5)	73 (37.8)	0.512
Use of statin (%)	227 (47.6)	134 (47.3%)	93 (47.9%)	0.829
Aortic arch calcification, score*	1.0 (0–2)	0.5 (0–1.5)	1 (0–2)	< 0.001

*Median with interquartile ranges. *SBP* systolic blood pressure, *DBP* diastolic blood pressure, *BMI* body mass index, Ca * P calcium phosphorus products, *HDL* high density lipoprotein, *LDL* low density lipoprotein, *BNP* B-natriuretic peptide, *hsCRP* high-sensitivity C-reactive protein.

Based on this, Table 1 compares the baseline characteristics of patients divided by the galectin-3 levels of 37.0 ng/mL. Patients with high galectin-3 levels were significantly older (p = 0.004), more likely to have a history of CAD (p = 0.021) and had higher diastolic BP (p = 0.027). Furthermore, WBC count (p < 0.001), peripheral neutrophil count (p < 0.001), NLR (p = 0.009), and serum inflammatory marker, hsCRP (p < 0.001) and BNP levels (p < 0.001) were significantly higher in the high galectin-3 group compared with the low group. However, serum Ca, p, and CaxP product levels were not different between the two groups. Interestingly, we found that patients with high galectin-3 levels had significantly lower serum HDL-C levels than patients with low galectin-3 levels, but total cholesterol, LDL-C, and triglyceride levels were comparable.

### Galectin-3 levels and aortic arch vascular calcification

**Figure 2:**
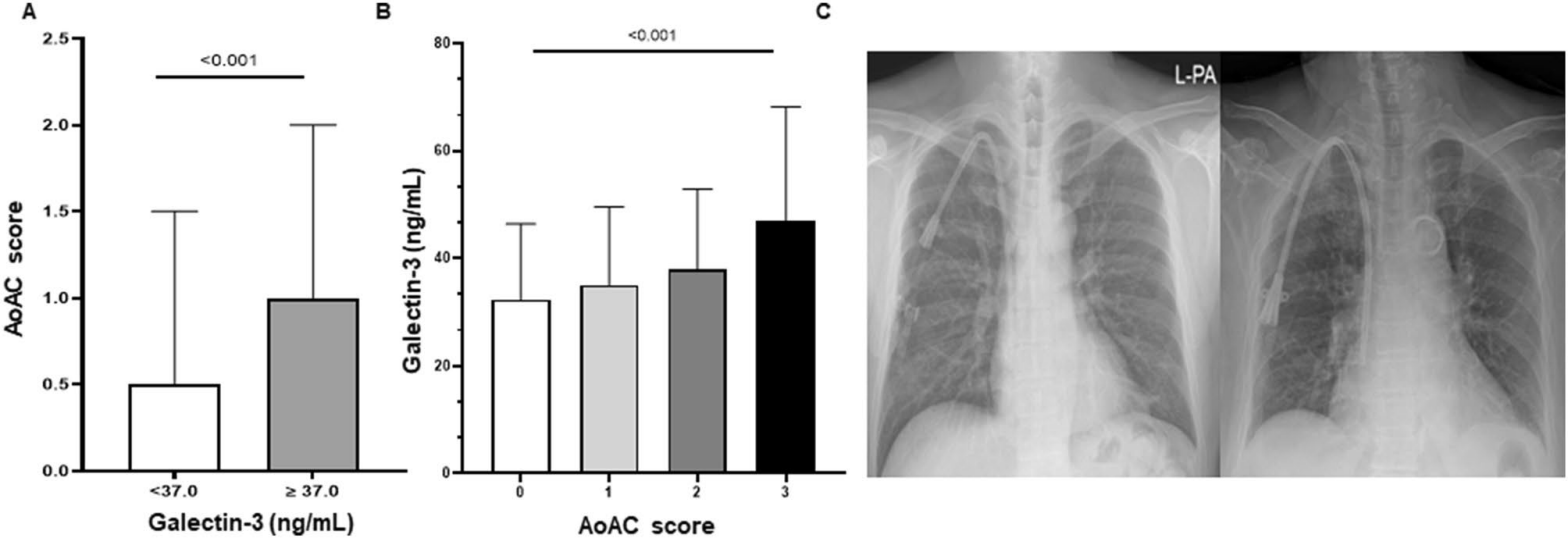
(**A**) Comparison of AoAC score in the two groups by serum galectin-3 level of 37.0 ng/mL. The AoAC score was higher in the groups with high galectin-3 (≥ 37.0 ng/mL) compared to the group with low galectin-3 (< 37.0 ng/mL) (p < 0.001). (**B**) Comparison of serum galectin-3 levels by AoAC scores. The higher the AoAC score, the higher the level of serum galectin-3 (p < 0.001). (**C**) Representative images of AoAC by galectin-3 level of 37.0 ng/mL. Left: grade 0, right: grade 3.

The prevalence of AoAC was 57% (n = 272). Among these patients, grade 1, 2, and 3 AoAC were observed in 118, 103, and 51 patients, respectively. We found that patients with higher galectin-3 levels had a significantly increased AoAC score; the median AoAC score was (with interquartile ranges) was 0.5 (0–1.5) and 1 (0–2) in patients with low and high serum galectin-3 levels, respectively (Table 1). Similarly, the higher the AoAC score, the higher the serum galectin-3 levels (p < 0.001) (Fig. 2A,B). Figure 2C shows representative images of AoAC by galectin-3 level. Correlation analysis showed a strong association between serum galectin-3 levels and age (r = 0.135, p = 0.003), CAD history (r = 0.117, p = 0.010), neutrophil count (r = 0.217, p < 0.001), serum BNP (r = 0.149, p = 0.006), ln-hsCRP levels (r = 0.306, p < 0.001), and AoAC score (r = 0.255, p < 0.001). And we also found a significant negative association between serum galectin-3 and HDL-C (r = − 0.181, p < 0.001) independent of the use of lipid-lowering therapy, whereas total cholesterol, LDL-C and triglyceride levels were not associated with galectin-3 levels (Table 2). With regard to the AoAC score, it was significantly associated with older age, higher galectin-3 levels, lower diastolic BP, and higher levels of hsCRP, neutrophils, and BNP. However, the AoAC score was not associated with lipid profiles. Multivariate logistic regression analysis showed that a 1-year increase in age and a galectin-3 level above 37 ng/mL were associated with a 9% (OR 1.09, 95% CI 1.06–1.12, p < 0.001) and twofold increased risk of VC (OR 1.99, 95% CI 1.15–3.42, p = 0.013), respectively (Table 3).

**Table 2:** Correlations between galectin-3, aortic arch calcification, and various vascular risk profiles.

	Age	SBP	DBP	Diabetes	CAD	Neutrophil	hsCRP	BNP	HDL	LDL	TG	AoAC
Galectin-3	0.135**	0.069	0.138*	0.028	0.117*	0.217**	0.306**	0.149**	− 0.181**	0.101	0.043	0.255**
Age		− 0.097	− 0.275**	− 0.001	0.160	0.009	0.207**	0.100	− 0.041	− 0.045	− 0.204**	0.527**
SBP			0.566**	0.243**	0.026	0.096	− 0.074	− 0.112	− 0.010	0.070	0.160	0.014
DBP				0.095	0.025	0.0780	0.069	− 0.137	− 0.008	0.080	0.04	− 0.188**
DM					0.121*	0.180**	0.085	0.070	− 0.135**	− 0.012	0.021	0.032
CAD						0.104*	0.122*	0.173**	− 0.128*	0.022	− 0.015	0.143**
Neutrophil count							0.274**	0.112	− 0.172*	0.034	0.033	0.145**
hsCRP								0.213**	− 0.163**	− 0.065	− 0.055	0.196**
BNP									0.036	0.095	− 0.101	0.125*
HDL										0.166**	− 0.247**	− 0.074
LDL											0.251**	− 0.015
TG												− 0.047

*p < 0.05, **p < 0.01. *SBP* systolic blood pressure, *DBP* diastolic blood pressure, *CAD* coronary artery disease, *HDL* high density lipoprotein, *LDL* low density lipoprotein, *BNP* B-natriuretic peptide, *hsCRP* high-sensitivity C-reactive protein, *AoAC* aortic arch calcification.

**Table 3:** Relationship between galectin-3 levels and aortic calcification.

	Vascular calcification (AoAC ≥ 1)			
	Univariate analysis		Multivariate analysis	
	OR (95% CI)	p	OR (95% CI)	p
Age	1.10 (1.07–1.12)	< 0.001	1.09 (1.06–1.12)	< 0.001
Sex, male	1.76 (1.21–2.58)	0.003	1.34 (0.78–2.30)	0.289
CAD, history	2.55 (1.56–4.18)	< 0.001	1.88 (0.97–3.65)	0.061
Diabetes	1.22 (0.84–1.78)	0.285	–	–
Calcium, mg/dL	1.03 (0.84–1.26)	0.777	–	–
Phosphate ≥ 5.0 mg/dL	0.81 (0.56–1.16)	0.255	–	–
Albumin < 3.5 g/dL	1.17 (0.79–1.73)	0.416	–	–
hsCRP ≥ 1.0 mg/L	1.63 (1.04–2.56)	0.033	1.09 (0.64–1.86)	0.746
HDL-C, mg/dL	0.99 (0.98–1.01)	0.706	–	–
Galectin-3 ≥ 37 ng/mL	2.06 (1.41–3.02)	< 0.001	1.99 (1.15–3.42)	0.013

*CAD* coronary artery disease, *hsCRP* high-sensitivity C-reactive protein, *BNP* B-natriuretic peptide, *HDL-C* high density lipoprotein-cholesterol.

### Galectin-3 levels and mortality

**Figure 3:**
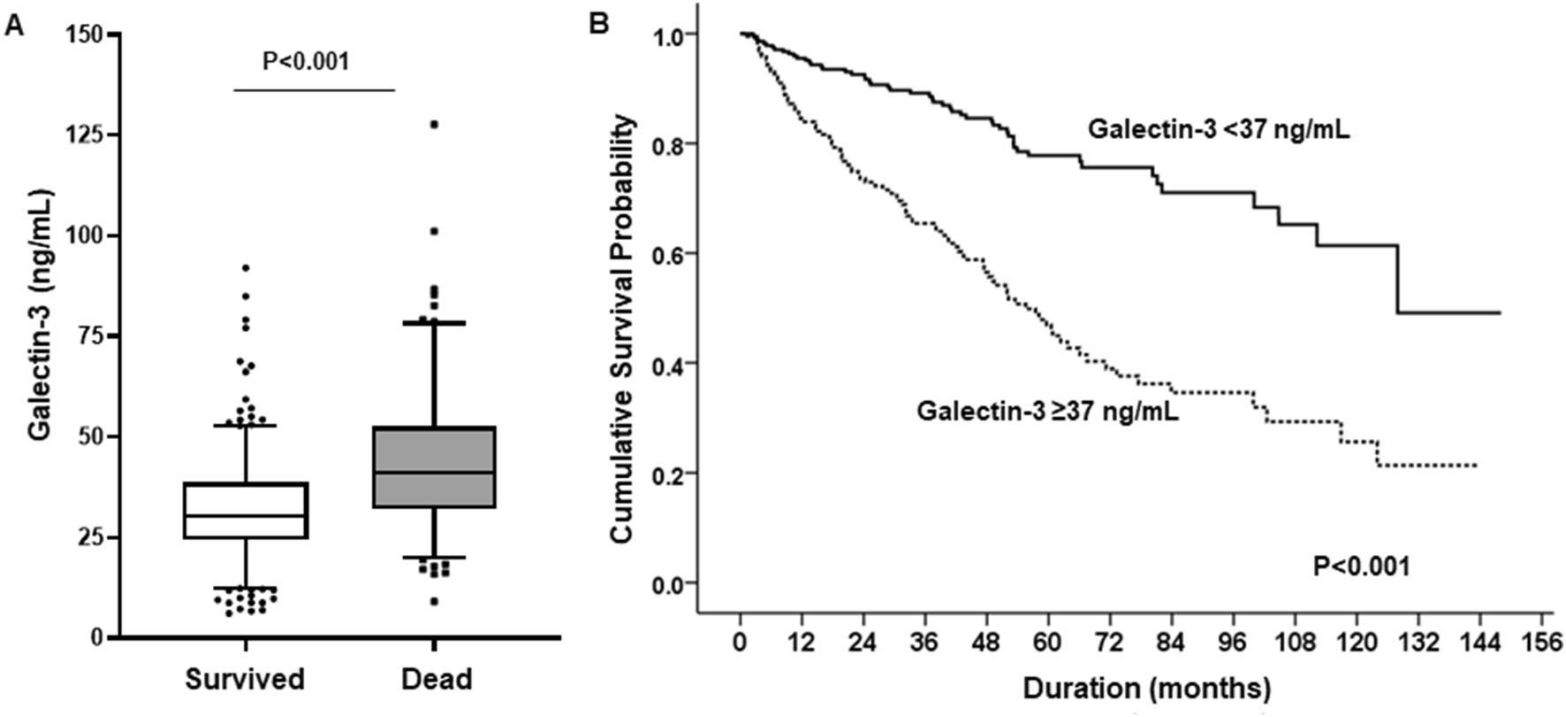
(**A**) Significant difference was observed in the serum galectin-3 levels in surviving and deceased HD patients (p < 0.001). (**B**) Kaplan–Meier survival analysis for mortality in the two groups by serum galectin-3 level of 37.0 ng/mL. Patients with galectin-3 ≥ 37.0 ng/mL had significantly higher mortality rates than those with lower levels.

During a median follow-up of 40 months (interquartile range: 16–63 months), 149 deaths (31.2%) occurred. The most common causes of death were CV events (n = 64), infections (n = 61), malignancy (n = 14), and other (n = 10). Baseline mean galectin-3 levels were significantly higher in patients who died than in those who survived (43.8 ± 17.8 vs. 32.2 ± 13.5, p < 0.001), and Kaplan–Meier survival analysis showed a significantly higher mortality rate in patients with galectin-3 ≥ 37.0 ng/mL than in those with lower levels (HR 3.08, 95% CI 2.20–4.32, p < 0.001) (Fig. 3A,B). In addition, older age, CVC use, history of CAD, hypoalbuminemia, high BNP level ≥ 1200 mmol/L, hsCRP level ≥ 1.0 mg/L, HDL-C, and AoAC were all associated with an increased risk of mortality in univariate Cox regression analysis. Even after adjustment for all risk factors, elevated galectin-3 levels remained significant predictors of all-cause mortality (HR 1.71, 95% CI 1.02–2.92, p = 0.048) (Table 4).

**Table 4:** Multivariate analysis predicting the risk of mortality with aortic calcification and higher galectin levels (≥ 37.0 ng/mL).

	All-cause mortality			
	Univariate analysis		Multivariate analysis*	
	HR (95% CI)	p	HR (95% CI)	p
Age	1.07 (1.05–1.09)	< 0.001	1.05 (1.02–1.07)	0.001
Sex, male	0.92 (0.66–1.28)	0.626	–	–
CVC use	3.26 (2.30–4.61)	< 0.001	3.27 (1.78–6.03)	< 0.001
CAD, history	1.60 (1.13–2.27)	0.008	0.73 (0.40–1.33)	0.301
Diabetes	1.03 (0.74–1.45)	0.860	–	–
Albumin < 3.5 g/dL	1.60 (1.14–2.24)	0.006	1.13 (0.67–1.89)	0.652
Phosphate ≥ 5.0 mg/dL	0.74 (0.53–1.03)	0.101	–	–
hsCRP ≥ 1.0 mg/L	2.32 (1.48–3.65)	< 0.001	1.49 (1.02–2.59)	0.046
HDL-C mg/dL	0.98 (0.97–0.99)	0.032	1.00 (0.98–1.02)	0.704
BNP ≥ 1200 mmol/L	1.67 (1.14–2.44)	0.008	1.27 (0.72–2.26)	0.410
Galectin-3 ≥ 37 ng/mL	3.08 (2.20–4.32)	< 0.001	1.71 (1.02–2.92)	0.048
AoAC				
0	Reference		Reference	
1	1.34 (0.83–2.16)	0.227	1.34 (0.65–2.78)	0.434
2	2.37 (1.51–3.73)	< 0.001	1.81 (0.92–3.91)	0.127
3	7.48 (4.81–11.62)	< 0.001	1.98 (1.01–4.00)	0.049

*CVC* central venous catheter, *CAD* coronary artery disease, *HDL* high density lipoprotein, *hsCRP* high-sensitivity C-reactive protein, *BNP* brain-natriuretic peptide, *AoAC* aortic arch calcification.

However, galectin-3 levels were not associated with cause-specific mortality, and there was no difference in galectin-3 levels by cause of death. The mean levels of galectin-3 in CV death, infection-related death, and malignancy-related death were 45.1 ± 19.0, 40.8 ± 15.6, and 53.2 ± 19.9 ng/mL, respectively (p = 0.755). In univariate analysis, higher galectin-3 levels were significantly associated with each cause of death: the HR for mortality from CV death, infection, and malignancy by galectin-3 level of 37 ng/mL was 3.78 (95% CI 2.22–6.42, p < 0.001), 2.29 (95% CI 1.37–3.81, p = 0.001), and 6.20 (1.72–22.2, p = 0.005), respectively. However, it was not statistically significant in multivariate analysis (Supplementary Table 2).

### Causal mediation

**Figure 4:**
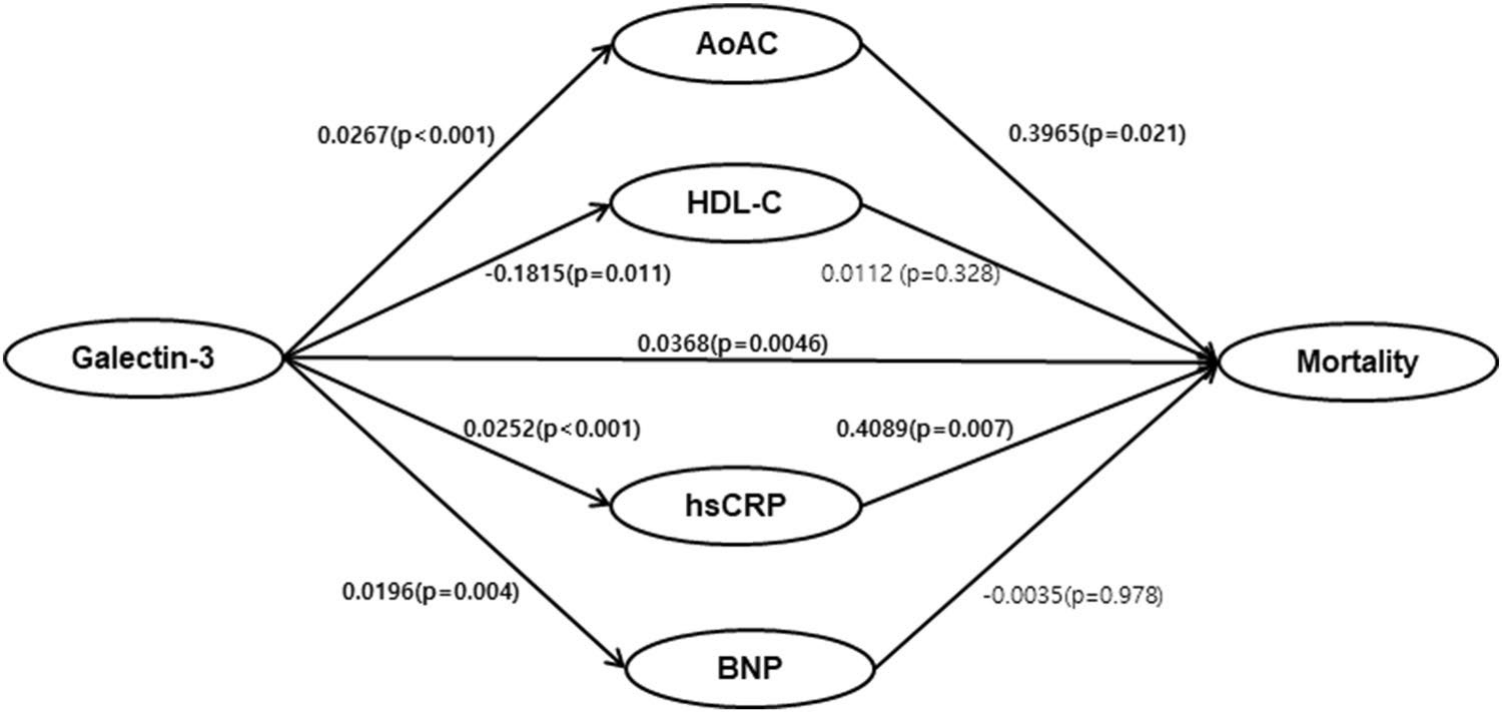
Results of mediation analysis. Both the direct effect of the galectin-3 on mortality (β = 0.0368, bootstrapped 95% CI [0.0113–0.0622]), and indirect effects through increased AoAC score (β = 0.0106, bootstrapped 95% CI [0.0015–0.0231]) and higher hsCRP levels (β = 0.0103, bootstrapped 95% CI [0.0028–0.0217]) were significant. BNP and HDL-C levels did not mediate the association between galectin-3 and mortality.

As we found that serum galectin-3 levels were closely associated with higher AoAC score, higher serum hsCRP, BNP, and low HDL-C levels, we hypothesized that galectin-3 might affect mortality through these factors. Results based on 5000 bootstrapped samples showed that both the direct effect of galectin-3 on mortality (β = 0.0368, bootstrapped 95% CI [0.0130–0.0622], p < 0.001) and the indirect effects were significant (total indirect effects: β = 0.0188, bootstrapped 95% CI [0.0066–0.0352]) (Table 5). There was a statistically significant indirect effect of galectin-3 on mortality via AoAC score (β = 0.0055, bootstrapped 95% CI [0.0015–0.0231]) and hsCRP levels (β = 0.0049, bootstrapped 95% CI [0.0028–0.0217]). However, BNP and HDL-C levels did not mediate the association between galectin-3 and mortality (Table 5 and Fig. 4). These data suggest that the significant effect of high galectin-3 levels on mortality is mediated via the induction of VC and the inflammatory state in HD patients.

**Table 5:** Mediation effects on the mortality.

Effects	Estimate	Bootstrapped 95% CI		
	β	SE	Lower limit	Upper limit
Indirect effect through AoAC	0.0106	0.0055	0.0015	0.0231
Indirect effect through hsCRP	0.0103	0.0049	0.0028	0.0217
Indirect effect through HDL-C	− 0.0020	0.0024	− 0.0077	0.0021
Indirect effect through BNP	− 0.0001	0.0030	− 0.0063	0.0060
Total indirect effect	0.0188	0.0073	0.0066	0.0352
Total direct effect	0.0368	0.0130	0.0113	0.0622

*SE* standard error, *CI* confidence interval, *HDL-C* high density lipoprotein cholesterol, *hsCRP* high-sensitivity C-reactive protein, *BNP* brain natriuretic peptide, *AoAC* aortic arch calcification.

## Discussion

The aim of this study was to investigate the causal relationship between high levels of galectin-3 and VC and all-cause mortality in ESRD patients who newly started on HD. First, we found that 57% of HD patients had AoAC at baseline, and higher serum galectin-3 levels were associated with an increased risk of VC. The more severe the AoAC score, the higher the galectin-3 levels were observed. In addition, the high galectin-3 levels significantly increased the risk of all-cause mortality. The mediation analysis showed that the significant effect of high galectin-3 levels on mortality was mediated by the induction of VC and inflammatory state.

Galectin-3, a soluble β-galactoside-binding lectin involved in cell proliferation, adhesion, migration and apoptosis, appears to promote inflammation or tissue fibrosis. Over the past decades, the prognostic role of serum galectin-3 as a biomarker linking oxidative stress, inflammation and fibrosis has been demonstrated in many pathological conditions such as HF, atherosclerosis, CKD and cancer^5,20–22^. Especially in patients with CKD, a recent large meta-analysis of 5226 patients showed that high galectin-3 levels were associated with an increased risk of all-cause mortality and CV events^10^. Similarly, in HD patients, Hogas et al. showed that a level of galectin-3 > 23.73 ng/mL was an independent predictor of mortality (HR: 2.60; 95% CI[1.09, 6.18])^23^, and a more long-term data also showed similar results^11^. Confirming a previous study, our data also showed that high galectin-3 ≥ 37.0 ng/mL was an independent predictor of mortality (HR 1.71, 95% CI 1.02–2.92, p = 0.048) even after adjustment of various CV risk factors.

However, galectin-3 levels were not associated with cause-specific mortality. In fact, there was no significant difference in galectin-3 levels by cause of death, and the prognostic role was consistent across cause-specific mortality. In univariate analysis, higher galectin-3 levels were significantly associated with each cause of death—CV death, infection-related death, and malignancy-related death, respectively. Although it lost statistical significance in multivariate analysis, we felt that this was due to the relatively small number of cases. We therefore believe that high galectin-3 levels are not a predictor limited to specific diseases, but are also associated with death from any cause, because galectins regulate basic cellular functions such as cell–cell and cell–matrix interactions, growth, proliferation, differentiation, and inflammation. It is therefore not surprising that this protein is associated with many causes of death^24^. In support of this, in the field of HF, galectin-3 has been shown to have diagnostic and prognostic value and has been recommended as a novel biological indicator for disease risk stratification of disease. In addition, abnormal galectin-3 expression is known to be associated with cancer initiation, progression, and metastasis^21^. In this context, galectins have become a focus of therapeutic research for clinical intervention against many pathological disorders^25,26^.

One of the most likely mechanisms linking galectin-3 and poor outcome is its proinflammatory properties. Galectin-3 is involved in macrophage chemotaxis, phagocytosis, neutrophil activation, oxidative stress and apoptosis, which are major pathogenesis of atherosclerosis^27^. In support of this, our data showed that serum galectin-3 levels correlated well with peripheral WBC count, circulating neutrophil count, NLR, and hsCRP levels, which are well-known markers of advanced atherosclerosis. This finding may indicate a strong link between galectin-3 and atherosclerosis via vascular inflammation.

Recently, it has also been suggested that galectin-3 may be involved in the osteogenic differentiation of VSMCs and the resultant development and progression of VC. As VC is a key feature of atherosclerosis, its early detection and treatment are of great importance for CV risk stratification and prevention. Especially in CKD, the burden of CV complications is significantly higher, which may be related to the widespread presence of VC in CKD patients. Our data showed that the higher the level of serum galectin-3, the more severe the degree of AoAC, providing clinical evidence that galectin-3 may play a role in VC in uremia. galectin-3 level above 37 ng/mL were associated with a twofold increased risk of VC. Other studies also showed the expression of galectin-3 at the site of calcified atherosclerotic plaque^28,29^, and Ibarrola et al. confirmed that inhibition of galectin-3 can reduce calcification of the heart valve in animal study^30^, suggesting the pathological role of galectin-3 in inducing VC.

In addition, very interestingly, we found negative association between galectin-3 and serum HDL-C levels. HDL-C is known as an antioxidant that plays a protective role against inflammation and oxidative stress in the human body^31,32^. Consistent with our data, previous data have reported that galectin-3 levels were negatively associated with HDL-C levels in the general population and in patients with MI^6^, suggesting that galectin-3 may be a link between dyslipidemia and inflammation. In addition, Zeng et al. reported that the combination of high galectin-3 and low HDL-C significantly improved the predictive value of recurrent stroke, and vascular events in patients with ischemic stroke^7^, suggesting that the benefit of the combination of the galectin-3 and HDL-C for predicting poor prognosis. In mechanism, both low HDL-C and high galectin-3 can induce lipid modification in oxidized LDL-C and enhance the phagocytosis of macrophages in taking up the oxidized LDL-C, exacerbating atherosclerosis and plaque rupture in vascular inflammation.

In this regard, we tried to find the mediation effect of various vascular risk factors on the relationship between galectin-3 and mortality. We found that AoAC and hsCRP significantly contribute to the overall indirect effect, however, BNP and HDL-C did not mediate the relationship. The results demonstrate the importance of VC and inflammatory status in mediating galectin-3 and all-cause mortality in HD patients.

Several limitations of our study should be mentioned. First, this is a single-center study with a retrospective analysis of a prospectively collected cohort. However, with a well-managed dialysis cohort, longitudinal follow-up data were available, which made it possible to obtain survival data and exact causes of death. So, with the mediation analysis, we can get a causal relationship between serum galectin-3 levels, AoAC, and mortality. Second, we could not get data on other VC scores such as AAC or coronary artery calcification. Because chest radiographs were performed in all patients, we used only the AoAC score. However, AoAC has been reported to correlate well with AAC or coronary artery calcium. Third, we did not investigate several factors that affect VC, such as vitamin K. However, we believe that this bias is minimal because it is unlikely that participants would choose not to take medications that affect vitamin K levels.

In summary, this study may suggest a potential causal relationship between serum galectin-3 and increased mortality in HD patients by providing evidence that galectin-3 increases VC and high inflammatory status.

### Supplementary Information

Supplementary Tables.

## Data Availability

All data generated or analyzed during this study are included in this article. Further enquiries can be directed to the corresponding author.
