# Supplementary material for: Mediating effect of vascular calcification in galectin-3-related mortality in hemodialysis patients
Source: Sci Rep. 2024 Jan 10;14:939. doi: 10.1038/s41598-024-51383-2 (PMC10776847; doi:10.1038/s41598-024-51383-2)
Supplement: Supplementary file 1 — Supplementary Tables. [file 41598_2024_51383_MOESM1_ESM.docx]

**Supplementary Table 1. Multivariate analysis evaluating the predicting role of AAC and higher galectin levels (≥37.0 ng/mL) on cause-specific mortality.**

|  | **CV mortality (n=64)** | |  | **Infection-associated mortality (n=61)** | |  | **Malignancy-associated mortality (n=14)** | |
| --- | --- | --- | --- | --- | --- | --- | --- | --- |
|  | **HR (95% CI)** | **P** |  | **HR (95% CI)** | **P** |  | **HR (95% CI)** | **P** |
| Age | 1.05 (1.02-1.10) | 0.003 |  | 1.04 (1.01-1.08) | 0.008 |  | 1.06 (0.97-1.15) | 0.396 |
| Sex, male | 1.81 (0.95-3.47) | 0.071 |  | 1.02 (0.54-2.0) | 0.896 |  | 1.44 (0.23-8.97) | 0.691 |
| CVC user | 2.60 (1.20-5.65) | 0.016 |  | 2.89 (1.36-6.17) | 0.006 |  | 8.74 (0.99-85.1) | 0.062 |
| Albumin <3.5 g/dL | 1.05 (0.54-2.09) | 0.769 |  | 1.09 (0.52-2.11) | 0.799 |  | 11.90 (1.001-140.9) | 0.048 |
| hsCRP **≥**1.0 mg/L | 1.66 (0.81-3.37) | 0.163 |  | 1.87 (1.01-3.81) | 0.043 |  | 10.76 (0.86-88.31) | 0.948 |
| **Galectin-3 ≥37ng/mL** | **1.52 (0.76-3.01)** | **0.231** |  | **1.76 (0.91-3.42)** | **0.093** |  | **5.23 (0.77-72.31)** | **0.156** |
| AoAC |  |  |  |  |  |  |  |  |
| 0 | Reference |  |  | reference |  |  | reference |  |
| 1 | 1.80 (0.77-4.18) | 0.170 |  | 1.13 (0.37-2.49) | 0.951 |  | 0.40 (0.37-4.24) | 0.445 |
| 2 | 1.85 (0.66-5.29) | 0.200 |  | 2.55 (1.03-5.49) | 0.033 |  | - | - |
| 3 | 2.79 (1.01-7.76) | 0.048 |  | 1.81 (0.24-2.89) | 0.744 |  | 0.54 (0.06-5.01) | 0.592 |

Abbreviations: OR, odds ratio; CI, confidence interval; CAD, coronary artery disease; HDL, high density lipoprotein; hsCRP, high-sensitivity C-reactive protein; AoAC, aortic arch calcification.

**Supplementary Table 2. Comparison of three levels of galectin-3 for predicting mortality**

| **Cut-off** | **36 ng/mL** | **37 ng/mL** | **38 ng/mL** |
| --- | --- | --- | --- |
| Sensitivity, % | 62.8 | 60.2 | 58.1 |
| Specificity, % | 72.1 | 74.7 | 76.1 |
| Accuracy, % | 69.8 | 71.1 | 69.4 |
| PPV, % | 53.3 | 56.3 | 58.8 |
| NPV, % | 78.4 | 77.5 | 76.4 |
